# Coma recovery scale-r: variability in the disorder of consciousness

**DOI:** 10.1186/s12883-015-0455-5

**Published:** 2015-10-08

**Authors:** MD. Cortese, F. Riganello, F. Arcuri, ME. Pugliese, LF. Lucca, G. Dolce, WG. Sannita

**Affiliations:** Institute S. Anna and RAN (Research in Advanced Rehabilitation), Crotone, Italy; Department of Neuroscience, Ophthalmology and Genetics, University of Genova, 3, Largo P. Daneo, 16132 Genova, Italy; Department of Psychiatry, State University of New York, Stony Brook, NY USA

**Keywords:** CRS-r, Disorders of consciousness, Variability, Vegetative state, Minimally conscious state

## Abstract

**Background:**

Despite evidence from neuroimaging research, diagnosis and early prognosis in the vegetative (VS/UWS) and minimally conscious (MCS) states still depend on the observation of clinical signs of responsiveness. Multiple testing has documented a systematic variability during the day in the incidence of established signs of responsiveness. Spontaneous fluctuations of the Coma Recovery Scale-revised (CRS-r) scores are conceivable.

**Methods:**

We retrospectively analyzed the CRS-r repeatedly administered to 7 VS/UWS and 12 MCS subjects undergoing systematic observation during a conventional 13 weeks. rehabilitation plan.

**Results:**

The CRS-r global, visual and auditory scores were found higher in the morning than at the afternoon administration in both VS/UWS and MCS subgroups over the entire period of observation. The probability for a VS/UWS subject of being classified as MCS at the morning testing at least once during the 13 weeks. observation was as high as 30 %, i.e., compatible with the reported misdiagnosis rate between the two clinical conditions.

**Conclusions:**

Multiple CRS-r testing is advisable to minimize the risk of misclassification; estimates of spontaneous variability could be used to characterize with greater accuracy patients with disorder of consciousness and possibly help optimize the rehabilitation plan.

## Background

Diagnosis and the early prognosis of subjects in the *vegetative state/unresponsive wakefulness syndrome* (VS/UWS) [1, 2] or *minimally conscious state* (MCS) [3–7] still depend on the clinical evaluation of responsiveness [4, 5, 8], while functional assessment by neuroimaging remains mostly limited to research [5, 9–15]. The diagnostic error between the VS/UWS and MCS reportedly still hovers up to 25–45 %, with possible implications in the patients’ prognosis, treatment, allocation of resources, etc. [3, 4, 16]. Clinical scales have been developed to provide the attending physician and therapist with reliable criteria of behavioral assessment and to allow more accurate evaluations [3, 16, 17]. Among these, the Coma Recovery Scale - revised (CRS-r) is an established and widely used tool [18, 19], with higher percentage of MCS subjects correctly diagnosed and better overall classification accuracy than the current clinical criteria [7].

The within/between subject (spontaneous) variability of the signs in use to evaluate brain responsiveness in VS/UWS and MCS is a possible critical issue adding to the existing controversy on their pathophysiological meaning [17, 20]. The visual pursuit response, a major CRS-r item recognizable in 70–80 % of subjects in MCS [9, 21, 22] and key marker of evolution from VS/UWS [23, 24], has been also reported with lower incidence (~20–30 %) in subjects otherwise diagnosed as VS/UWS [20, 25–28]. Multiple testing has documented systematic spontaneous fluctuations in the incidence of this response in the course of the day in both VS/UWS and MCS subjects; the overall chance of observing it at least once per day was ~33 % and ~62 % in the VS/UWS and MCS subgroups, respectively, with maxima at 10.30 am and 3.00 pm and no response at postprandial time in both subjects’ groups [29]. Individual variability may suggest limited diagnostic accuracy for the visual pursuit response and, by extension, risk of erroneous CRS-r classification when subjects are tested at a single time point of the day. Purpose of this study was to analyze the spontaneous fluctuation over time of the CRS-r scores.

## Methods

We studied retrospectively the CRS-r scores obtained from two subgroups of subjects with disorder of consciousness undergoing a standard rehabilitation plan. They had been diagnosed by the attending physician as being in chronic VS/UWS (*n* = 7; age 51.8 ± 11.6 years.) or MCS (*n* = 12; age 51.4 ± 17.4 years.) at the beginning of treatment; diagnosis was based on the current clinical criteria and guidelines by the Aspen group [19]. The CRS-r global score was equal or lower than 7 (mean = 6.42 ± 0.49) in VS/UWS subjects and between 9 and 15 in the MCS patients (mean = 11.08 ± 1.75). Subjects clinically unstable, under treatment with (neuro)active drugs or beta-blockers, with anoxia, history of psychiatric disorder, concurrent systemic diseases, or evidence of recurrent pain were not considered. Subjects requiring Arousal Facilitation Protocol procedures (AFP) were also excluded in order to avoid the interference of these procedures on their responses to the CRS-r stimulus condition. The VS/UWS and MCS subjects’ subgroups did not differ as to age (Mann-Whitney’s test: z = −0.466, *p* > 0.05) or length of hospitalization (1144.3 ± 551.2 days in VS/UWS and 1711.4 ± 455 days in MCS; z = −1945, *p* > 0.05). Summary demographics and clinical information are reported in Tables 1 and 2.

**Table 1:** Summary demographics and clinical information at the beginning of the rehabilitation plan. (The patients’ relatives and caregivers gave their consent to the use of the patients’ data)

	Sex	Age range	Aetiology	CRS-r			Time from brain injury (days)
				Total score	Visual subscore	Auditory subscore	
VS/UWS	Female	38–73	Vascular	6	1	1	662
			Other	7	0	1	2278
			Vascular	6	1	1	663
			Vascular	7	0	1	958
			Vascular	6	0	2	1256
	Male	41–65	Traumatic	6	1	1	1199
			Traumatic	7	1	1	1201
MCS	Male	31–65	Vascular	9	3	2	1199
			Traumatic	10	3	2	1492
			Traumatic	10	3	0	1958
			Traumatic	13	3	1	2380
			Traumatic	11	2	1	2455
			Traumatic	11	2	1	2345
			Traumatic	15	3	3	2399
	Female	35–79	Traumatic	9	3	2	2251
			Vascular	10	3	1	1393
			Vascular	10	3	1	1773
			Vascular	13	2	1	2298
			Traumatic	12	3	1	1542

**Table 2:** Summary demographics and clinical information at the beginning of the rehabilitation plan. (The patients’ relatives and caregivers gave their consent to the use of the patients’ data)

Score	Auditory scale	Visual scale	Motor scale	Oromotor/Verbal scale	Communication scale	Arousal scale
6	-	-	Functional Object Use	-	-	-
5	-	Object Recognition	Automatic Motor Response	-	-	-
4	Consistent movement to command	Object Localization: Reaching	Object Manipulation	-	-	-
3	Reproducible Movement to Command	Visual Pursuit	Localization to Noxious Stimulation	Intelligible Verbalization	-	Attention
2	Localization to Sound	Fixation	Flexion Withdrawal	Vocalization/Oral Movement	Functional: Accurate	Eye Opening w/o Stimulation
1	Auditory Startle	Visual Startle	Abnormal Posturing	Oral Reflexive Movement	Non-Functional: Intentional	Eye Opening with Stimulation
0	None	None	None/Flaccid	None	None	Unarousable

The study has been approved by the local public health care Ethical Committee (Provincial Health Authority of Crotone). The patients’ relatives and caregivers were informed about the study aims and gave their consent to the use of the patients’ data, which have been always treated under condition of anonymity. The ethical principles of the Declaration of Helsinki (1964) by the World Medical Association concerning human experimentation were followed.

Patients were treated following a standard plan with alternating 2 weeks. periods of intensive rehabilitation (two sessions/day, morning and afternoon; hereafter Phase A) and 1 week. periods of standard rehabilitation (one session/day; Phase B) for a total of 13 weeks. The protocol began and ended with the phase B (Fig. 2). Subjects received nursing care before 9.00 a.m. and after 5.00 p.m. and were fed at noon compliant to the unit schedule; the rehabilitation sessions and CRS-r evaluation always began at least 30 min. after the morning nursing care in order to avoid induced arousal or other possible interference.

CRS-r was always administered before rehabilitation and in the two time windows of the day when responsiveness had previously proved highest in a previous study (9.30–11.00 a.m.; 3.00–4.00 p.m.) [29]. CRS-r administration was once a week in phases A (in the morning the first week and in the afternoon the second week); in phases B, it was administered in the morning and afternoon at the beginning and end of the week. The CRS-r was administered following the international guidelines by two expert examiners (a neuropsychologist and an occupational therapist) who were requested to examine each patient together and to reach an agreement on each measured item. When tested, patients were comfortably sitting on an armchair in a familiar setting with a constant temperature of 24 °C in the absence of environmental noise or interference; in no case, patients were reported to have showed discomfort in the presence of the examiners or during testing. Only patients with matching numbers of assessments were considered in the study. Scores from 342 CRS-r administrations were obtained in total (18 times per subject).

The CRS-r global scores and subscores at the morning and afternoon ratings and at the beginning and end of the protocol were compared. Statistical processing was by Wilcoxon signed-rank test, after collapsing the data from each patient into a pair of values to avoid alpha inflation due to the sample size when comparing conditions (morning vs afternoon; baseline vs. end of treatment). To this end, nine values from the morning assessment and the corresponding nine values at the afternoon administration (4 from phase A and 5 from phase B) were averaged. The effect size (*r*) (i.e., the index measuring the magnitude of difference or change between two conditions, in this case baseline vs. end of the protocol and morning vs. afternoon) [30] was calculated as the z/square root (N) (where N is the number of observations on which z is based) and will be hereafter formally referred to as not relevant (*r* < 0.1), small (0.1 < r < 0.3), medium (0.3 < r < 0.5), or large (*r* > 0.5) [31]. The Bonferroni correction for multiple testing was applied for a *p* < 0.005 level of statistical significance on ten comparisons between morning and afternoon. The risk of misclassification of subjects in VS/UWS or MCS to the other clinical condition when relying only on a single (morning or afternoon) assessment was tested by the Odds Ratio and Risk Ratio in both subjects’ groups.

## Results and discussion

**Fig. 1:**
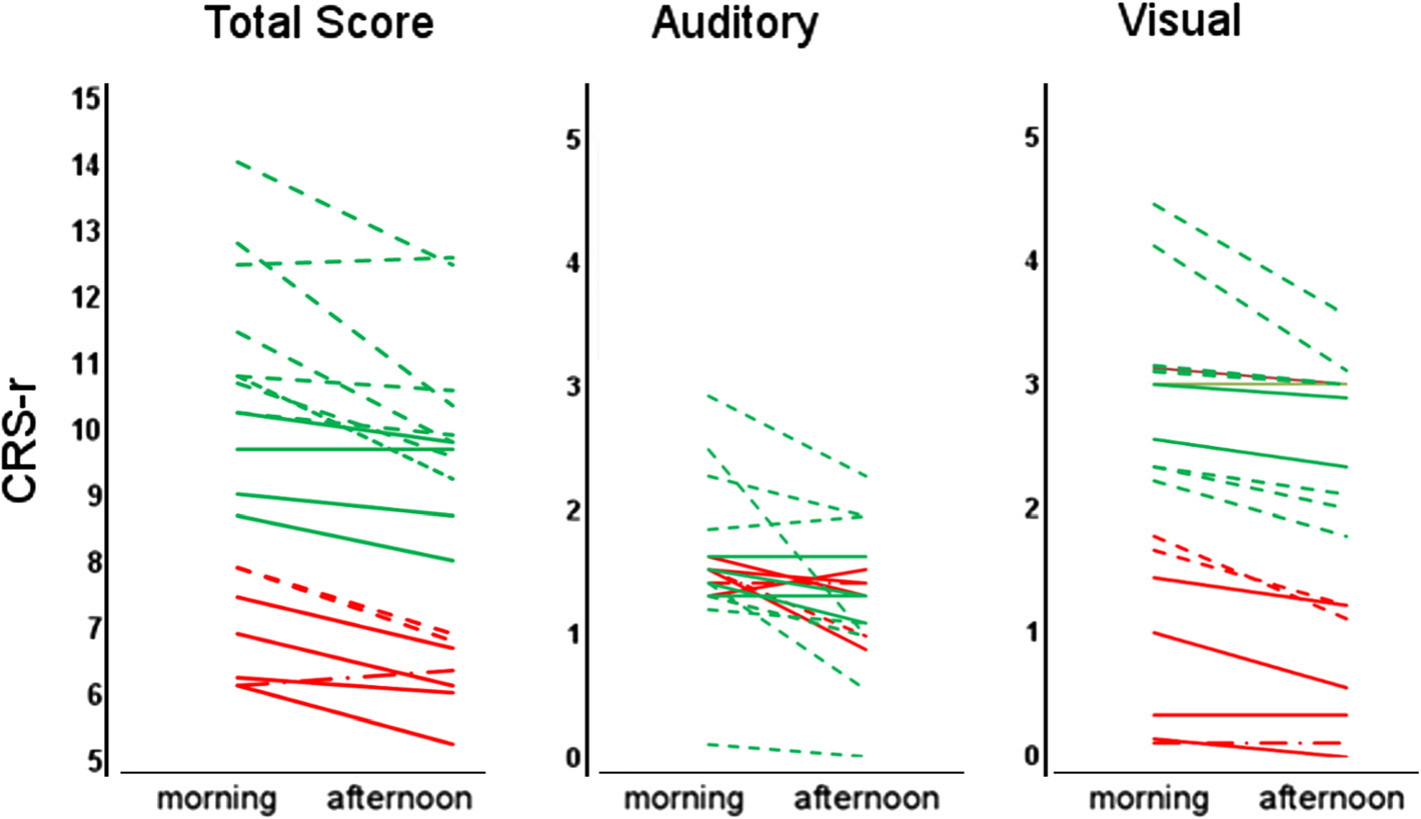
CRS-r total and visual and auditory scores (means of 9 values at the morning and 9 at the afternoon assessments) for each VS/UWS (*red*) or MCS (*green*) subject. Dashed: vascular; continous: traumatic; dash-line: other etiologies

**Fig. 2:**
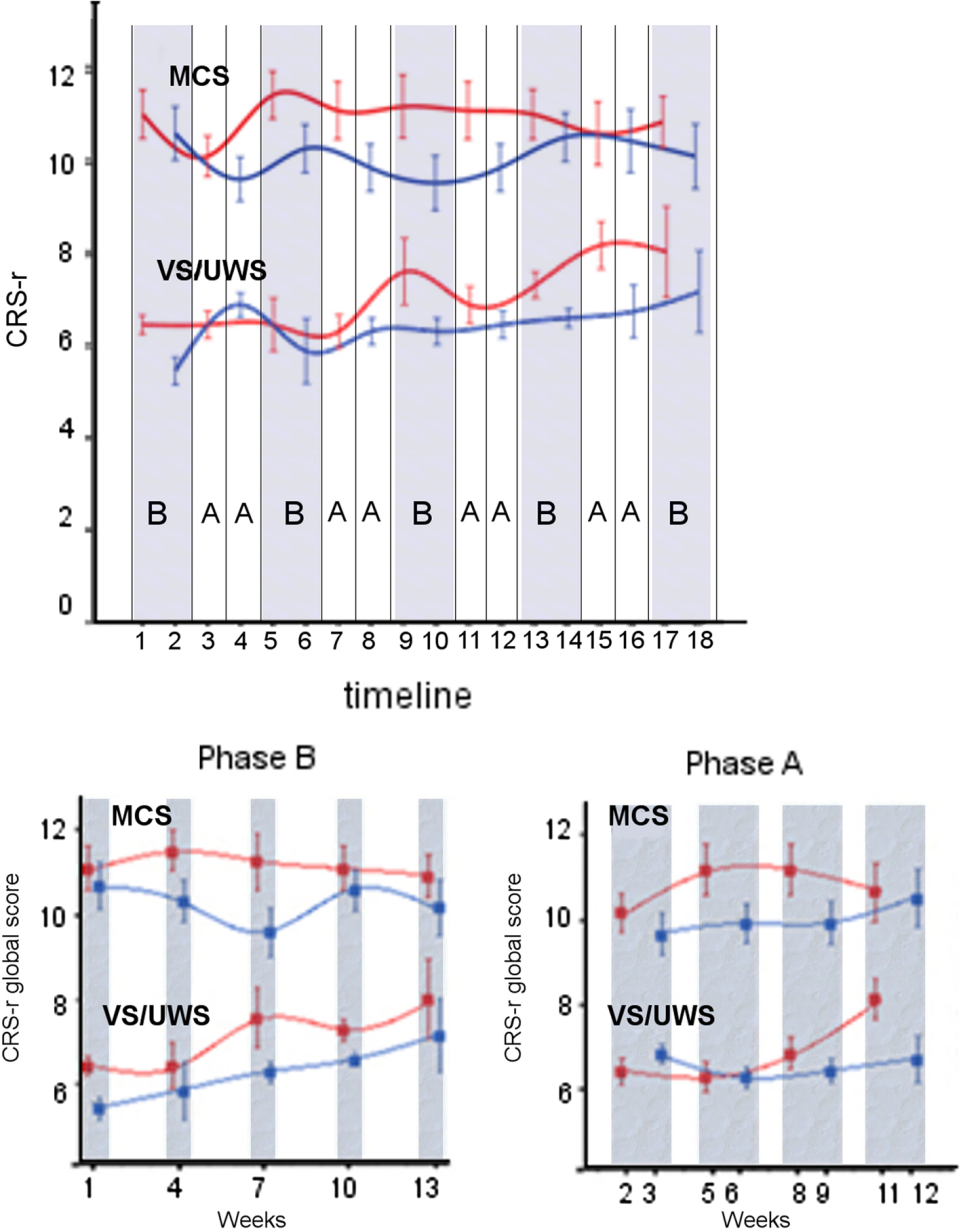
Top: mean and SE across subjects of the CRS-r global score at the morning (*red*) and afternoon (*blue*) testing in VS/UWS and MCS. Bottom: CRS-r global scores (mean, SE) at the morning (*red*) and afternoon (*blue*) testing in the two treatment phases A and B

**Fig. 3:**
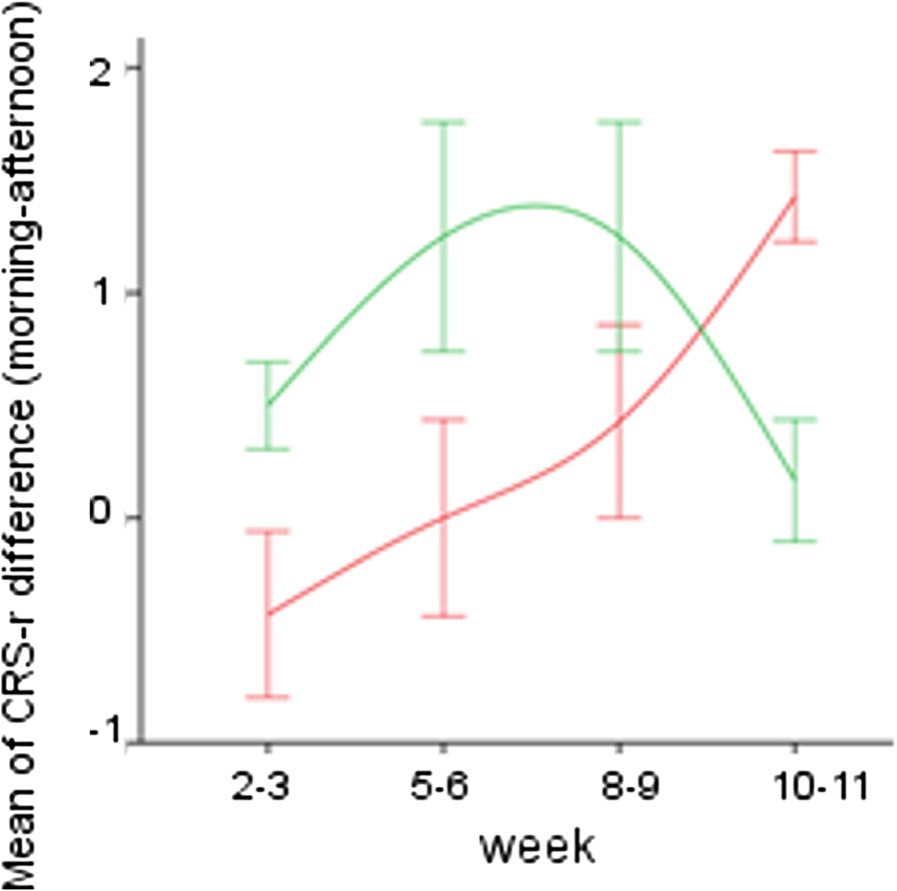
Mean difference (morning minus afternoon) and SE of the CRS-r global scores in VS/UWS subjects (*red*) and MCS (*green*). Positive values indicate CRS-r scores higher at the morning

Individual CRS-r scores at the morning and afternoon scale administration are shown in Fig. 1. In both VS/UWS and MCS subgroups and over the entire period of observation, the mean CRS-r global scores were higher at the morning assessment (7 ± 1.5 and 11 ± 1.9 for VS/UWS and MCS, respectively) than in the afternoon (6.3 ± 1.3 and 10.1 ± 1.9) (Wilcoxon’s z = −3.916, *p* < 0.0001, *r* = 1.04 and z = −5.195, *p* < 0.0001, *r* = 1.06) (Fig. 2). Scores were higher in the morning in both Phases A and B in the MCS subgroup (Wilcoxon’s z = −3.513, p[correct] < 0.001, *r* = 0.75 and z = −3.843, p[correct] = 0.018, *r* = 0.82, respectively), but only in Phase B the difference reached statistical significance in the VS/UWS subgroup (Wilcoxon’s z = −3.840, p[correct] < 0.001, *r* = 1.02). The morning vs. afternoon difference was observed also when considering posttraumatic subjects or patients with vascular brain injury separately (Wilcoxon’s z = 2.874, p[correct] = 0.024, *r* = 0.67 and Wilcoxon’ = s z = −4.415, p[correct] = 0.000, *r* = 0.75 respectively) (Fig. 1). It increased progressively over the 13 weeks. observation period in VS/UWS, while reaching a maximum between the 6^th^ and 9^th^ weeks of protocol to decline thereafter in the MCS subgroup (Figs. 2 and 3); as a result, the mean CRS-r global scores were higher at the end of the protocol compared to baseline in the VS/UWS (7.6 ± 2.4 and 5.9 ± 0.8, respectively; morning-afternoon average; Wilcoxon’s z: −2.347, p = 0.019, effect size: *r* = 0.63), but not in MCS (Figs. 2 and 3). The morning vs. afternoon difference in global score was accounted for by the visual and auditory items (Fig. 1) both in the MCS (Wilcoxon’s z = −3.062, p correct = 0.01, *r* = 0.65 and z = −3.721, p correct < 0.001, *r* = 0.79) and VS/UWS subjects’ groups (Wilcoxon’s z = −2.874, p correct = 0.02, *r* = 0.77 and z = −2.744, p correct = 0.03, *r* = 0.73). No significant contributions from the motor, oromotor/verbal, communication and arousal subscales to the differences in the CRS-r global score were observed.

The measure of association in VS/UWS subjects between the CRS-r scores at the morning or afternoon assessments and the risk of wrong attribution to the MCS condition was significant (odds ratio = 4.75; 95 % confidence interval: 1.73–12.7; *p* = 0.001; relative risk =1.4; 95 % coefficient interval: 1.15–1.7; *p* = 0.001). The risk was higher in the phases B (odds ratio = 5.09; 95 % confidence interval: 1.38–18.7; *p* = 0.014; relative risk =1.76; 95 % coefficient interval: 1.30–1.19; *p* = 0.014) than in phases A (odds ratio = 1.70; 95 % confidence interval: 10.51–2.55; *p* = 0.5; relative risk =0.78; 95 % coefficient interval: 2.04–1.86; *p* = 0.014). The risk of wrong classification was lower in MCS group (odds ratio = 0.49; 95 % confidence interval: 0.087–2.73; *p* = 0.44; relative risk =0.66; 95 % coefficient interval: 0.21–2.06; *p* = 0.44). The estimated probability of observing during the rehabilitation protocol CRS-r items compatible with MCS in subjects diagnosed as VS/UWS was 30 % at the morning (range: 0–55 %) and 9.5 % at the afternoon assessments (range: 0–22 %).

These observations derive from a retrospective analysis of the CRS-r scores obtained during a conventional rehabilitation protocol that had not been designed to test differences in the subjects’ responsiveness. In this respect, the differences between the protocol phases A and B or the possible effects of intensive treatment are not suitable of detailed investigation or pathophysiological interpretation. Prospective studies are needed for this purpose. This caveat notwithstanding, the results both invite speculation and suggest caution in the use of the available clinical assessment tools. The CRS-r was administered at the same two time windows during the day when responsiveness had previously proved highest in chronic VS/UWS and MCS subjects [29]. The global score and visual and auditory subscores nevertheless proved higher in the morning than in the afternoon, with the mean differences in the CRS-r global score increasing with time in the VS/UWS subjects’ subgroup; the morning vs. afternoon difference was about twice larger than the average improvement at the end of the protocol (54.2 % vs. 28.8 %) which confirms the large size effect estimated statistically. The variability of the CRS-r assessment was unrelated to etiology in our patients’ sample, but a relationship with etiology cannot be ruled out in principle.

A positive visual pursuit response (a major CRS-r item) results of activation in the anterior and posterior midline structures of the brain (mesiofrontal and precuneal cortices) [32–35], which are metabolically impaired in the severe disorder of consciousness according to neuroimaging studies [36, 37]. Neuroimaging has also documented regional activation in response to stimulus conditions in VS/UWS [12, 14, 20, 38, 39]; responses to stimulus conditions purported to induce emotional reaction have been described [40–42]. Whether any of these responses may indicate “automatic” subcortical processing atypical for the VS/UWS or it marks higher order cortical activation and partially recovered consciousness, remains an unsolved controversy [19]. Yet, the observation of responses such as these is conventionally regarded as indicative of surviving modules of the corticocortical and brainstem-cortex functional interaction that is thought to sustain consciousness in the awake subject [6, 43–48] and is interfered with in the VS/UWS and MCS [2, 9, 10, 19, 21, 22, 36, 37, 39, 49, 50]. In this respect, the CRS-r reliability (as also documented by the relationship between EEG descriptors and CRS-r scores) [51] is not to be questioned on the ground of its variability during the day, nor is to be questioned the examiners’ accuracy. Instead, the observed CRS-r differences between morning and afternoon are likely to reflect individual changes in the subject’s level of visual, auditory and motor functioning conceivably due to changes in the neuronal/non-neuronal factors that modulate the brain state [52, 53]. Spontaneous fluctuations of any of these factors may be expected to result in random differences in responsiveness rather than in systematic difference during the day; effects of fragmentary circadian/ultradian or otherwise cyclic processes (e.g., in metabolism) are thus also conceivable, albeit not documentable in the absence of multiple assessments at short intervals over the 24 h. period [22].

The extent to which the within-day variability in the CRS-r global, visual and auditory scores observed in this study may have clinical relevance in the diagnosis and early prognosis of subjects with disorder of consciousness remains to be studied in large patients’ samples. Systematic investigation on the spontaneous variability over time of the relevant neuronal or non-neuronal parameters in the severe disorder of consciousness is also advisable and may help characterize these conditions in greater detail [52]. The possible source(s) of variability notwithstanding, the estimated risk of misclassification for a single random CRS-r testing appears compatible with the reported misdiagnosis between VS/UWS and MCS [3, 4, 6, 7].

## Conclusions

Multiple CRS-r assessments at different times of the day and monitoring over time are advisable. Diagnostic criteria taking also individual variability into a proper account may help reduce misdiagnosis between conditions sharing the underlying pathophysiology, but differing as to clinical picture, prognosis, required medical care and logistics, legal or popular perception of bioethical issues, allocated resources, healthcare policies, etc. [1, 6, 40, 54–56]. These considerations aside, the evidence of higher responsiveness in the morning compared to the afternoon invites discussion on whether the time window dedicated to treatment is irrelevant to rehabilitation or it should be planned accordingly.

## Abbreviations

VS/UWS: Vegetative state/unresponsive wakefulness syndrome
MCS: Minimally conscious state
CRS-r: Coma recovery scale-revised
SE: Standard error
